# Overall cognitive profiles in patients with GLUT1 Deficiency Syndrome

**DOI:** 10.1002/brb3.1224

**Published:** 2019-02-04

**Authors:** Valentina De Giorgis, Silvia Masnada, Costanza Varesio, Matteo A. Chiappedi, Martina Zanaboni, Ludovica Pasca, Melissa Filippini, Joyce A. Macasaet, Marialuisa Valente, Cinzia Ferraris, Anna Tagliabue, Pierangelo Veggiotti

**Affiliations:** ^1^ Department of Child Neurology and Psychiatry IRCCS Mondino Foundation Pavia Italy; ^2^ Department of Brain and Behavioral Sciences University of Pavia Pavia Italy; ^3^ Brain and Behavior Department University of Pavia Pavia Italy; ^4^ Child Neurology Unit IRCCS Istituto delle Scienze Neurologiche Bologna Italy; ^5^ Department of Neurosciences Makati Medical Center Manila Philippines; ^6^ Genomic and post‐Genomic Center IRCCS ‘‘C. Mondino’’ National Neurological Institute Pavia Italy; ^7^ Human Nutrition and Eating Disorder Research Center Department of Public Health Experimental and Forensic Medicine University of Pavia Pavia Italy; ^8^ Pediatric Neurology Unit “V. Buzzi” Hospital Milan Italy; ^9^ Biomedical and Clinical Sciences Department, L Sacco University of Milan Milan Italy

**Keywords:** cognitive profile, epilepsy, GLUT1DS, ketogenic diet, movement disorder

## Abstract

**Introduction:**

Glucose Transporter Type I Deficiency Syndrome (GLUT1DS) classical symptoms are seizures, involuntary movements, and cognitive impairment but so far the literature has not devoted much attention to the last.

**Methods:**

In our retrospective study involving 25 patients with established GLUT1DS diagnosis, we describe the cognitive impairment of these patients in detail and their response to the ketogenic diet in terms of cognitive improvement.

**Results:**

We outlined a specific cognitive profile where performance skills were more affected than verbal ones, with prominent deficiencies in visuospatial and visuomotor abilities. We demonstrated the efficacy of ketogenic diet (KD) on cognitive outcome, with particular improvement tin total and verbal IQ; we found that timing of KD introduction was inversely related to IQ outcome: the later the starting of KD, the lower the IQ, more notable nonverbal scale (verbal IQ correlation coefficient −0.634, *p*‐value = 0.015). We found a significant direct correlation between cognition and CSF/blood glucose ratio values: the higher the ratio, the better the cognitive improvement in response to diet (from T0–baseline evaluation to T1 on average 18 months after introduction of KD‐: TIQ correlation coefficient 0.592, *p*‐value = 0.26; VIQ correlation coefficient 0.555, *p*‐value = 0.039). Finally, we demonstrated that a longer duration of treatment is necessary to find an improvement in patients with “severely low ratio.”

**Conclusion:**

Our results were consistent with the hypothesis that timing of the diet introduction is a predictive factor of cognitive outcome in these patients, confirming that earlier initiation of the diet may prevent the onset of all GLUT1DS symptoms: epilepsy, movement disorders, and cognitive impairment.

## 1 INTRODUCTION

Glucose Transporter Type I Deficiency Syndrome (GLUT1DS) is a metabolic disorder due to mutations in SLC2A1 gene (1p 31.3→35) (Shows et al., 1987) which encodes for a specific transporter of glucose in the brain. Ketogenic diet (KD), which provides ketone bodies instead of D‐glucose as alternative fuel for cerebral metabolism, is at current time the gold standard therapy for GLUT1DS (Klepper, 2008).

Since the first description of the disease by De Vivo et al. (1991), different studies have led to identification of “common manifestations” of this disease (microcephaly, cognitive impairment, epilepsy, continuous movement disorders, and paroxysmal exercise‐induced dyskinesia) and “uncommon manifestations” (paroxysmal kinesigenic dyskinesia [PKD], paroxysmal nonkinesigenic dyskinesia [PNKD], oculogyric crises, and fatigue) that suggest a high probability of GLUT1DS as a clinical diagnosis (De Giorgis, Varesio, Baldassari, Olivotto, & Veggiotti, 2016).

Epilepsy occurs in about 90% of cases and seizure types vary widely as follows in order of frequency: generalized tonic‐clonic seizures (GTCs), absence, complex partial, myoclonic, drop attacks, tonic, simple partial, infantile spasms, and epileptic spasms (Pong et al., 2012). KD was proven to have an anticonvulsant property in many studies (Kass, Winesett, Bessone, Turner, & Kossoff, 2016; Leen et al., 2010; Pong et al., 2012; Ramm‐Pettersen et al., 2013) but the underlying mechanism is not completely understood (Clanton, Wu, Akabani, & Aramayo, 2017; Politi, Shemer‐Meiri, Shuper, & Aharoni, 2011).

In addition to seizures, involuntary movements are common symptoms of GLUT1DS (De Giorgis et al., 2016) which also demonstrated a positive response to KD, (Leen et al., 2010; Veggiotti et al., 2010), even if improvement was less evident compared to the effect on seizures.

Regarding cognitive impairment in GLUT1DS patients, several reports describe a mild or moderate‐severe mental disability (without a clear definition of degree), in most cases proportional to disease's severity (Hully et al., 2015; Larsen et al., 2015; Leen et al., 2010; Tzadok et al., 2014) (for an overview of literature see Table 1). Hully et al. (2015) observed that patients with myoclonic seizures have a higher likelihood of cognitive impairment. Ito et al. (2015) found that mental disability was more severe in patients with earlier disease onset; furthermore, they found a better cognitive outcome in patients with missense mutations, higher CSF/blood glucose ratio, and a later age of disease onset.

**Table 1:** Cognition in GLUT1DS: overview on literature

Paper	Total N	Data available	Intellectual disability (before KD)					Other Neuropsychiatric symptoms	KD cognitive and behavioral improvement
			n° (%) ID patients	Severe	Moderate	Mild/Borderline	ID unclassifiable		Pre/post
Klepper (2008)	15	15	15 (100%)				15		Parents reported an increase of alertness and activity (mean KD duration 36 months)
Coman et al. (2006)	8	8	8 (100%)		1	1	6		1 pt more alert and interactive (KD duration 6 weeks)
Suls et al. (2009)	4	4	3 (75%)		1	1 mild, 1 borderline			
Akman et al. (2010)	13	13	11 (85%)	1		5 mild, 5 borderline			
Leen et al. (2010)	57	54	54 (98%)	8	21	24	1		(KD duration not available) 21/44 pt improvement of cognitive functions, alertness, and behavior
Ito et al. (2011)	6	6	6 (100%)	4	1	1			MAD (Modified Atkins Diet) (mean KD duration 19.6 months) Pt1: IQ 65/67; Pt2: IQ 33/41; Pt3: IQ 30/35; Pt4: IQ 48/50 Improvement in vigilance and motivation (6/6), comprehension (5/6), concentration (4/6)
Ramm‐Pettersen et al. (2013)	10	9	6 (67%)		2	2 mild	1, 1 learning disability	1 pt no eye contact	(mean KD duration 31.9 months available for 8/10 patients) Pt3: developmental delay, no eye contact/maj,or improvement Pt4: moderately/slightly ID Pt5: moderate ID/learning disability Pt6: learning disability/improved endurance Pt10: NA/more alert, improve endurance
Leen et al. (2010)	7	7	7 (100%)	1	2	4			(mean KD duration 90 months) 6/7 pt, positive effects on cognition
Ragona et al. (2014)	1	1	1 (100%)			1 borderline IQ		ADHD, visuospatial and verbal memory	
Ramm‐Pettersen et al. (2014)	6	6	5 (83%)		2	1	2		(duration 6–17 months) KD Pt2: Bayley III Scaled Scores 5/WPPSI‐III IQ 102; Pt3: WPPSI‐III IQ 42/63; Pt4: WISC‐IV IQ 49/54 MAD Pt6: WAIS IQ 53/53 All Pts displayed an Improvement in alertness, language, social engagement, articulation, and physical endurance
Tzadok et al., 2014;	17	8	3 (43%)			1 mild	2 learning disability	2 ADHD 1 behavioral problems	(KD and MAD duration not available) 2 pt: Clinical impression of cognitive improvement
Alter et al. (2015)	13	13	12 (92%)		1	1	10	9 exceptional empathy, 1 impulsivity and inattentiveness	(duration 8.8 years) KD 7/7 on KD no improvement in cognitive functions; Ravens Colored Matrices mean 78.27/83.64
De Giorgis et al. (2015)	22	22	21 (95.5%)		5	6 mild, 10 borderline			(KD duration not available) KD 5/13 (37%) on KD improvement in cognitive functions, alertness, and activity Pt 12: WISC IQ 79/89
Ito et al. (2015)	57	33	33 (100%)	12	9	12		24% learning disabilities and ADHD, Pts had social and friendly personalities	
Hully et al. (2015)	58	24	22 (92%)	5	12	5		80% friendly disposition, excessive communicative and jovial behavior (12/24), impulsivity and hyperactivity (13/24)	(KD duration not available) 15 pt 68% improvement in cognitive functions and behavior
Larsen et al. (2015)	6	6	4 (67%)				4		
Total	300	229	211 (92.1%)	31 (14.7%)	57 (27%)	81 (38.4%)	42 (19.9%)		

On the other hand, in literature, no specific cognitive profiles of GLUT1DS patients are reported, except for a case report by Ragona et al. (2014) who described the natural evolution of cognitive profile of a patient in a span of 6 years follow‐up without KD. This patient presented a mild cognitive decline (8 years old: TIQ (total intelligence quotient): 95, VIQ (verbal intelligence quotient): 99, PIQ (performance intelligence quotient): 92; at 12 years old IQ: 84, VIQ: 88, and PIQ: 83) associated with an impairment on neuropsychological functions (attention, executive functions, visuospatial, and verbal memory) (Ragona et al., 2014).

Efficacy of KD on cognitive functions has been poorly investigated so far, even if an improvement was reported in terms of visuomotor precision (3/6 patients in Ramm‐Pettersen, Stabell, Nakken, & Selmer 2014), alertness/vigilance, and motivation (6/6 patients in Ito, Oguni, Ito, Oguni, & Osawa 2011; 5/13 patients in De Giorgis et al. 2015), IQ performance (2/6 patients in Ramm‐Pettersen et al. 2014), both expressive and receptive language (3/6 patients in Ramm‐Pettersen et al., 2014), and sensorimotor speed (1/6 patients in Ramm‐Pettersen et al. 2014). Total IQ improvement was found in 1/13 patients in an Italian group (De Giorgis et al., 2015) and in 4/6 patients in a Japanese population (Ito et al., 2011). Younger patients demonstrated the most noteworthy response on KD (Ramm‐Pettersen et al., 2013).

Duration of KD was also mentioned to have an impact on cognition, particularly with an improvement of TIQ. In Ramm‐Pettersen et al. 2014 after 14 months of KD, a patient gained 21 points of TIQ from 42 to 63 on Wechsler Preschool and Primary Scale of Intelligence (WPPSI III) (Wechsler, 2008); a patient in De Giorgis et al. 2015, after 2 years of diet, gained 10 points of TIQ from 79 to 89 on WISC III. Besides these rare reports, the cognitive profile in GLUT1DS has not been deeply characterized so far. Effectiveness of the diet on cognition is probably difficult to assess because of the presence of other genetic and environmental factors that could be involved in the outcome.

The aim of our study was to describe the cognitive profile in GLUT1DS patients, before and after the KD introduction, in order to define a specific cognitive profile—in terms of trend of specific indexes of Wechsler Intelligence Scales (total, verbal, and non verbal) and individual subtests—to correlate it to GLUT1 phenotype and outcome after KD introduction.

## 2 METHODS

### 2.1 Patient selection

This is a retrospective study involving 25 patients with established diagnosis of GLUT1DS, aged 3.7–40 years (mean 13.16), composed of seven males and 18 females. All patients were regularly followed up at Fondazione Istituto Neurologico Nazionale C. Mondino (Pavia, Italy) between 2007 and 2016. Informed consent was obtained from children's parents and patients. The study was approved by the Ethics Committee of our Institute.

For each patient included in the study, information such as type of GLUT1 mutation, cerebrospinal fluid (CSF)/blood glucose ratio, type of seizure, type of movement disorder, intelligence quotient (IQ) [total (TIQ), verbal (VIQ), and performance (PIQ)], and response to the KD were collected. Classical KD was given in 4:1, 3:1, or 2:1 ratio (grams of fat: carbohydrates plus proteins) in order to obtain beta‐hydroxybutyrate levels between 2 and 6 mmol/L in each patient.

### 2.2 Follow‐up evaluations

Baseline (T0) evaluations, referring to the time of GLUT1DS diagnosis, were available for all 25 patients. Data were collected at specific time intervals while on KD: T1, mean of 18 months (range 11–28), available in 14 patients (five patients dropped out and six patients did not reach T1 follow‐up at the time of the study); T2, mean of 36 months (range 27–48), available in six patients at the time of the study.

### 2.3 Neuropsychological assessment

Standard cognitive tests measured with Wechsler Intelligence Scales (Wechsler, 1995, 2012) according to the age of the patient were conducted at T0, T1, and T2. Test administration was carried out individually by a professional neuropsychologist. Testing was divided into two sessions; neither exceeded 45 min per subject per session. Cognitive function was expressed as Total Intelligence Quotient (TIQ), Verbal Intelligence Quotient (VIQ), and Performance Intelligence Quotient (PIQ).

Each subtest was analyzed to a have a clinical picture of cognitive function and its domain.

### 2.4 Statistical analyses

Statistical analysis was performed using SPSS statistical software version 19.0 for Windows (SPSS Inc., Chicago, IL). After testing for normal distribution by mean of the Kolmogorov–Smirnov test, we applied nonparametric tests. Matched data were compared with Wilcoxon signed rank test, while differences between groups were assessed using the Mann–Whitney *U*‐test. Values were expressed as medians and ranges, while categorical variables were described as absolute numbers and percentages.

Correlation analysis was then used to identify potential influencing factors for IQ amelioration in the whole sample. Nonparametric correlation coefficient (Spearman's Rho) was used, considering the presence of non‐normally distributed variables.

Clinical variables analyzed in relation to cognition were the presence and type of mutation (missense, nonsense, splice site, deletion, or frame shift), CSF/blood glucose ratio, and patient's age at the time of KD implementation.

### 2.5 Ethical statement

We confirm that we have read the Journal's position on issues involved in ethical publication and affirm that this report is consistent with those guidelines.

## 3 RESULTS

Our sample (presented in detail in Table 2) includes 25 patients (seven males and 18 females) aged between 3.7 and 40 years (mean 13.16 years), with established diagnosis of GLUT1 deficiency syndrome. De Giorgis et al. (2015) previously reported the clinical data of 22 patients, as presented in detail in Table 2.

**Table 2:** Clinical characteristics of GLUT1DS population

ID patient	Sex	Age at diagnosis	Mutation		CSF/blood	Epilepsy		Movement disorder		Other symptoms	KD	KD efficacy	
				Type	Ratio	Y/N	Type	Y/N	Type		Age	Epilepsy	MD
1	F	4 years	p.Arg153Cys	Missense	0.39	y	DS GTC ABS	y	C A	/	52	y	y
2	M	9.5 years	p.Pro485Leu	Missense	0.47	y	DS	y	PED C	W Mi	129	y	n
3	F	9 years	p.Arg126Cys	Missense	0.34	y	MAS DS ABS	y	PED	Ds W	119	y	y
4	M	11.5 years	p.Arg223Trp	Missense	0.51	y	CFS	y	PED C D	M	146	/	y
5	F	13 years	p.Pro36Arg	Missense	0.44	y	DS	y	C A	Ds	161	y	n
6	F	6.5 years	p.Val165IIe	Missense	0.38	n	/	y	PED D C	/	84	n	y
7	F	7 years	p.Asn34Cys	Missense	0.35	y	ABS	n	/	/	84	y	/
8	F	19 years	p.Gln283Ter	Nonsense	0.33	y	FS ABS GTC MS	y	C A	Ds	235	y	y
9	M	7.5 years	C1257delG_1delG SPL	Spice site	0.54	y	GTC ABS	y	PED D	W	96	y	y
10	F	18 years	Trp48Term	Nonsense	0.33	y	ABS GTC MS	y	C A	Ds W Mi	221	y	y
11	F	20 years	p.Arg126Cys	Missense	0, 38	y	ABS FS MS	y	C A	Ds W Mi	245	y	y
12	F	15 years	p.Arg153Cys	Missense	0.56	y	GTC FS	y	PED D	Mi	189	/	y
13	F	8.5 years	p.Arg400cys	missense	0.38	y	ABSMAS	y	PED D		112	y	y
14	M	14.5 years	p.Arg400His	Missense	0.44	n	/	y	PED D M	O Mi Ds	180	n	y
15	F	13.5 years	Thr295Met	Missense	0.42	y	ABS	y	PED D	/	169	y	y
16	F	30 years	p.Val165IIe	Missense	0.44	y	ABS GTC	y	PED	/	NA	/	/
17	F	19 years	p.Va1166del	Deletion	0.5	y	ABS	y	PED D	O Mi	233	y	y
18	F	6.5 years	p.Leu124TrpfsX12	Deletion	0.36	y	MS	n	/	/	84	y	/
19	M	18 years	p.Arg458Trp	Missense	0.51	y	CFS	y	PED D	Ds	223	y	y
20	M	38 years	p.Asn34Ser	Missense	0.43	y	ABS	y	PED D	/	NA	/	/
21	M	9 years	p.Leu67Pro	Missense	0.5	n	/	y	PED C	/	118	/	y
22	F	9.5 years	p.Thr9Met	Missense	0.52	y	ABS	n	/	/	122	y	/
23	F	9 years	p.Gly398Ser	Missense	0.73	y	CFS MS ABS	n	/	/	111	y	/
24	F	1.5 years	p.ARg249Ala fs*131	Deletion	0.37	y	MAS	n	/	/	22	y	/
25	F	16 years	p.Ala275Thr	Missense	NA	y	ABS	/	/	Mi	NA	/	/

F, female; M, male; Ratio, CSF/blood glucose ratio; NA, not available; Y, yes; N, no; DS, dyscognitive seizures; ABS, absence seizure; GTC generalised tonic‐clonic seizure; FS, febrile seizures; MAS, myoclonic astatic seizures; CSF, complex febrile seizures; PED paroxismal exertion‐induced dyskinesia; A ataxia; D, dystonia, C, choreoatetosis, M, myoclonias; W, weakness; Mi, migraine; Ds, dysarthria; O, Oculogiric Crises; KD, ketogenic diet; Age, months; MD, movement disorders. KD efficacy refers to seizure freedom and disappearance of movement disorders.

Mutational findings were, in order of frequency, missense mutation (19 patients), nonsense mutation (two patients), deletion (two patients), frame shift mutation (one patient), and splice site mutation (one patient).

The mean CSF/blood glucose ratio was 0.5 (range 0.34–0.73).

The majority of patients (22 of 25) were treated with classical KD (4:1, 3:1, or 2:1 fat to nonfat ratio) with an adequate compliance and no serious side effects reported. Diet was introduced at a mean age of 142 months (range 22–245 months). Epilepsy had a positive response to KD in 81% (17 of 21 are seizure free) and involuntary movements resolved in 84% (16 of 19).

Intelligence quotient scores measured by Wechsler Intelligence Scales were available in all patients at T0 (detailed description of results in Supplementary Table A). The median scores were: total IQ of 61 (range: 40–99, IQR (interquartile range): 29), VIQ of 66 (range: 45–118; IQR: 38), and PIQ of 68 (range: 45–98; IQR: 32).

Stratification of patients according to their mental disability showed:

- Five patients with normal TIQ (mean 95.4; range 91–99)
- Six patients with borderline TIQ (mean 78; range 74–84)
- Seven patients with mild cognitive impairment (mean 57.57; range 51–66)
- Seven patients with moderate‐severe cognitive impairment (mean TIQ 45.14; range 40–50)

A discrepancy in standard scores (differences of 10, 20, 40 points between VIQ and PIQ with lower PIQ score than VIQ) was present in 80% (20/25) of our subjects. In particular 40% (12 patients) had a discrepancy of <10 points, 20% (five patients) had a discrepancy >10 points, and 12% (three patients) had a discrepancy of >20 points (one patient had >40 standard scores discrepancy). Meanwhile, patients with mild cognitive impairment presented at the opposite, having lower VIQ scores than PIQ in 50% of the patients.

Dividing the subjects into four groups according to the TIQ, we found that discrepancy of >10 and >20 points was present uniformly in the all groups: (two in normal TIQ, two in borderline TIQ; one in the mild cognitive impairment, and three in the moderate cognitive impairment).

Taking into consideration PIQ, the specific subtests(detailed description of results in Supplementary Table B) that were mostly affected were Picture Completion with a median standard score of 5 (range: 1–8; IQR: 5); Coding‐Digit Symbol with a median standard score 4.5 (range: 1–9; IQR: 3); Picture Arrangement with a median standard score of 3 (range: 1–9; IQR: 1); and Block design with a median standard score of 5 (range: 1–9; IQR: 4.75).

### 3.1 KD follow‐up evaluations (T1, 18 months)

**Figure 1:**
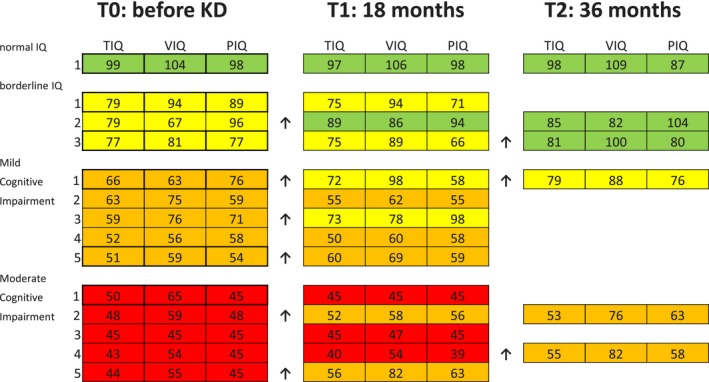
IQ (Intelligence Quotient) scores at different time points during the follow‐up. In green Normal IQ, in yellow Borderline IQ, in orange Mild Cognitive impairment (ID) and in red moderate Cognitive impairment. The arrows point out the patients that had an IQ amelioration passing from moderate IQ to Mild IQ, or from Mild IQ to Borderline IQ, or from Borderline IQ to Normal IQ

Cognitive data after 18 months of follow‐up (T1) were available in 14 patients. Among these, median IQ scores varied with an improvement in TIQ from 55.5 (range: 43–99; IQR: 25.75) to 58 (range: 40–97; IQR: 24), VIQ from 64 (range: 45–104; IQR: 19) to 73.5 (range: 45–106; IQR: 29.75) and PIQ was substantially stationary (from 58.5 [range: 45–98; IQR: 31] to 58.5 [range: 45–98; IQR: 14.5]). To cite, one patient progressed from borderline to normal IQ, two patients slightly improved from mild cognitive impairment to borderline IQ and two patients improved significantly from moderate to mild cognitive impairment (for more details see Figure 1).

Correlation between IQ evolution (total, verbal, and performance) and type of mutation was attempted, but we did not find any statistically significant result, likewise for type of seizure and movement disorder.

Instead, correlation between CSF/blood glucose ratio and IQ (total, verbal and performance) showed an improvement of TIQ and VIQ from T0 to T1 among patients with higher CSF/blood glucose ratio (TIQ correlation coefficient 0.592, *p*‐value = 0.026; VIQ correlation coefficient 0.555, *p*‐value = 0.039).

**Figure 2:**
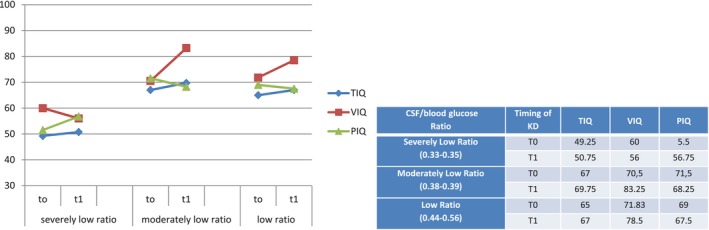
Age group according to Ketogenic Diet (KD) initiation and its corresponding WISC‐III Scores (Total Intelligence Quotient TIQ, Verbal Intelligence Quotient VIQ, Performance Intelligence Quotient PIQ) at T0 (Baseline) and at T1 (18th month on KD). Early age group (four patients), mean 79 months; Middle age group (eight patients), mean 144 months; Older age group (three patients), mean 233 months at the age of KD implementation

On the base of CSF/blood glucose ratio, we classified patients into three groups as “low ratio” group (>0.40), “moderately low ratio” group (0.36–0.39), and “severely low ratio” group (≤ 0.35). IQ (total, verbal and performance) evolution in relation to CSF/blood glucose ratio was as follows:

- in “severely low ratio” group TIQ was substantially stationary with worsening of VIQ and an improvement in PIQ.
- in “moderately low” and “low ratio” groups TIQ and VIQ improved over time while PIQ worsened (Figure 2).

Timing of KD introduction was inversely related to IQ outcome: the older the patient, the lower the IQ, in a more evident way on verbal scale (VIQ correlation coefficient −0.634, *p*‐value = 0.015).

**Figure 3:**
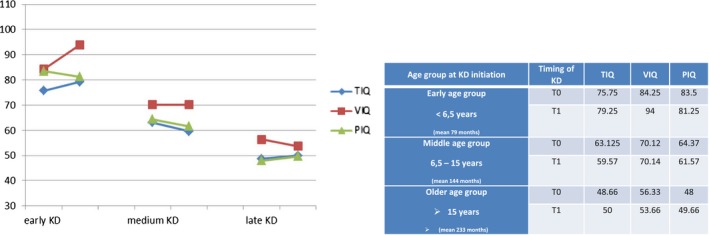
CSF/blood glucose ratio and its corresponding WISC‐III Scores at T0 (baseline) and at T1 (18th month of KD). Severely Low Ratio (0.33–0.35) (three patients); Moderately Low Ratio (0.38–0.39) (four patients); Low Ratio (0.44–0.56) (six patients)

As shown in Figure 3 patients who received KD earlier had a better IQ at T0 and improved further. Dividing our patients into three groups according to the age of KD implementation we found that younger patients (mean 6.6 years) had better cognitive outcome (in terms of TIQ, VIQ, and PIQ); middle age group (mean 12 years) showed a stable verbal IQ with a worsening in TIQ and PIQ; older age group (mean 19.5 years) acquired the lowest IQ scores (TIQ, VIQ and PIQ) at T1.

### 3.2 KD follow‐up evaluations (36 months, T2)

**Figure 4:**
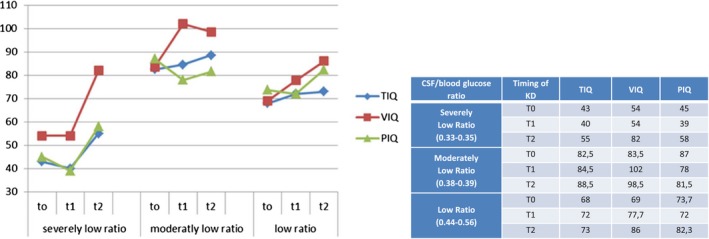
CSF/blood glucose ratio and its corresponding WISC‐III Scores at T0 (baseline), T1 (18th month of KD) and T2 (36th month of KD). Severely Low Ratio (0.33–0.35) (one patient); Moderately Low Ratio (0.38–0.39) (two patients); Low Ratio (0.44–0.56) (three patients)

Six patients were able to continue up to 36 months of KD treatment (T2). All demonstrated an improvement in all Intelligence Quotient domains (TIQ, VIQ, and PIQ). Moreover, stratifying the patients by CSF/blood glucose ratio as above, we found that those with “severely low ratio” did not show an improvement of the IQ in the short‐term follow‐up (T1), but a marked improvement was noted after a long‐term (T2) follow‐up (Figure 4) particularly in verbal scores (VIQ +28 points).

## 4 DISCUSSION

So far, most literature data focused on GLUT1DS general clinical profile in both pediatric and adult populations with predominantly qualitative description of cognitive function. The majority of reports try to correlate KD response with general outcome of al lGLUT1DS symptoms and describe cognitive and behavioral aspects when available.

Our experience clearly demonstrated a peculiar cognitive profile in GLUT1DS patients where performance IQ was more affected than verbal IQ. This result was prominent in patients with normal and borderline IQ, where a greater impairment in performance domain was found.

Analyzing each Wechsler Intelligence Scales (Wechsler, 1995, 2012) subtest, we identified a major impairment in performances such as Completion subtest, Coding‐Digit Symbol, Picture Arrangement, and Block design. Low scores in Picture Completion subtest reveals an impairment in attention capacity, ability to observe details, and recognize specific features of the environment. Low scores in Coding‐Digit Symbol imply an impairment in visual motor speed, motor coordination, memory, and visual analysis. Deficient scores in Picture Arrangement pertain to impairments in nonverbal reasoning, sequencing skills, temporospatial analysis, mental anticipation, planning capacity, speed, accuracy, and grasp of social cause and effect (also known as social intelligence). Lastly, deficient scores in Block Design reveal impairments in visual perceptual skills, speed, spatial problem‐solving, manipulative abilities, coordination, and fluid intelligence.

Considering typical clinical profile of GLUT1DS patients with prominent extrapyramidal symptoms such as motor incoordination, dysarthria, fatigue, continuous and paroxystic movement disorders, we speculate that hindrance in performance skills could be considerably influenced by this symptomatology. Even if KD improved performance and writing skills (Veggiotti et al., 2010), the response to KD on involuntary movements was not excellent (patients #2 #5).

Taking into account IQ lower scores in subtests described above, we can speculate that GLUT1DS patients have a typical cognitive profile with greater difficulties in visuospatial and visuomotor skills. Therefore, according to this evidence, we suggest a complete neuropsychological investigation in order to provide a protocol that better defines nonverbal, visuomotor, speed and accuracy of GLUT1DS patients.

In our study, we attempted to relate cognitive profile with clinical and genetic characteristics of GLUT1DS patients. We noticed that there is no direct correlation between type of mutation and cognitive impairment, as was noted by Ito et al. (2015); nor with type of movement disorder or type of seizure as observed by Hully et al. (2015).

On the other hand, we found a significant direct correlation between IQ (particularly TIQ and VIQ) and CSF/blood glucose ratio values in short‐term follow‐up (T1). Higher CSF/blood glucose ratio corresponded to a better cognitive improvement in response to KD measured at 18 months. This was clearly demonstrated by dividing our population into three groups according to their CSF/blood glucose ratio. In our sample, we observed a better cognitive outcome in patients with “low” and “moderately low” ratios compared to “severely low ratio” group. The “severely low ratio” group obtained an improvement in cognition, but a longer duration of treatment was necessary (T2).

Considering these results, we can infer that a longer duration of KD treatment may be necessary to compensate greater CNS glucose transporter defect, especially in patients with severely low‐ratio values.

In a longitudinal study, Alter et al. (2015) found that patients treated early in infancy had a better long‐term outcome. This group hypothesized that there could be a “window of vulnerability” where an increase in cerebral glucose metabolism, not balanced because of typical energy deficit found in GLUT1DS, causes damage to the immature brain. They placed the vulnerability period between first and sixth months after birth, so they stated that diagnosis and treatment in this window are critical for improved neurological outcome.

Although our population did not include patients with very early diagnosis and, thus, introduction of the KD in our sample was at an average age of 6 years, our results confirmed that the later the age of KD introduction, the worse the outcome of VIQ in both short and medium term of the diet.

Those patients who started diet before 6 years of age achieved a better cognitive outcome; those who started between 6 and 12 years had a moderate decline of total IQ (with a stable VIQ and worse PIQ). GLUT1DS patients who started KD in adolescence suffered the worst cognitive evolution.

Our results are consistent with the hypothesis that timing of KD introduction is a predictive factor for cognitive outcome in patients with GLUT1DS and that an earlier introduction of the diet may prevent not only epilepsy and movement disorder onset (previously widely demonstrated by different studies (Kass et al., 2016; Leen et al., 2010; Pong et al., 2012; Ramm‐Pettersen et al., 2013; Veggiotti et al., 2010) but also cognitive impairment. Furthermore, on the basis of our experience, we can speculate that the “window of vulnerability” could be expanded in the early childhood as far as cognition is concerned.

Although duration of dietary therapy was not clearly stated in GLUT1DS therapeutic guidelines, physicians agree about obvious efficacy and good tolerability of KD on epilepsy and involuntary movements in the long term. So far, less data are available in literature about cognition, but studies available showed a better cognitive outcome in patients with earlier diagnosis and early introduction of KD (Ramm‐Pettersen et al., 2013). Our data confirm that an early KD introduction and a good compliance to the diet are predictive also of a better cognitive outcome, and our study confirms that prolonged treatment with KD is needed in order to increase the chance of achieving at least a partial recovery of neuropsychological deficits. Other possible environmental factors may have influenced the cognitive improvements described in our patients: family care, speech and other supportive therapies, KD ratio and compliance to the KD; probably KD with higher ratios could give a more rapid and effective response. In our experience, patients with drastic diets, carried out for a long period had higher noncompliance issues and dropouts. For this reason, all our patients were prescribed a classical KD with variable ratios—but more frequently 3:1 or 2:1—with the aim of maintaining beta‐hydroxybutyrate levels between 2 and 6 mmol/L which have guaranteed the compliance to all of the patients recruited in our study and allowed a good efficacy response in terms of epilepsy, movement disorder, and cognition.

Considering that our results refer to a group of GLUT1DS patients with heterogeneous socio‐economic levels (parents’ education level, parents’ careers and salary, parents’ health) which influenced compliance to KD and cognitive outcome, it cannot be excluded that other environmental or unknown genetic factors could influence both initial cognitive competence and outcome after treatment. Definitely, other studies involving a larger population are needed to confirm our findings, characterize in more detail GLUT1DS patients’ cognitive profile and clearly assess response to KD therapy.

Based on our cognitive results, we suggest applying to all GLUT1DS patients a complete neuropsychological investigation, studying in detail the visuospatial and visuomotor skills which were more compromised in our sample.

## 5 LIMITATIONS

Future studies, which include a larger population, a longer follow‐up and higher statistical power, are necessary in order to obtain a better explanation of our results.

A relevant limitation of our study was the age of introduction of the KD conditioned by late diagnosis, so our data could not accurately outline the “window of vulnerability.”

## CONFLICTS OF INTEREST

Prof. Veggiotti Pierangelo has received speaker's fee from Eisai and Nutricia. The remaining authors have no conflicts of interest.
