# Supplementary material for: Overall cognitive profiles in patients with GLUT1 Deficiency Syndrome
Source: Brain Behav. 2019 Feb 4;9(3):e01224. doi: 10.1002/brb3.1224 (PMC6422708; doi:10.1002/brb3.1224)
Supplement: Supplementary file 1 [file BRB3-9-e01224-s001.pdf]

Supplementary Table A. IQ profiles of GLUT1DS population

ID	T0				T1				T2			
**patient**	age	TIQ	VIQ	PIQ	follow-up	TIQ	VIQ	PIQ	follow-up	TIQ	VIQ	PIQ
**1**	65	99	104	98	15	97	106	98	27	98	109	87
**2**	120	79	67	96	24	89	86	94	43	85	82	104
**3**	118	43	54	45	18	40	54	39	28	55	82	58
**4**	123	77	81	77	22	75	89	66	48	81	100	80
**5**	161	48	59	48	12	52	58	56	43	53	76	63
**6**	102	66	63	76	14	72	98	58	28	79	88	76
**7**	84	59	76	71	15	73	78	98				
**8**	235	45	45	45	16	45	47	45				
**9**	133	79	94	89	14	75	94	71				
**10**	221	50	65	45	25	45	45	45				
**11**	245	51	59	54	11	60	69	59				
**12**	144	63	75	59	20	55	62	55				
**13**	131	52	56	58	28	50	60	58				
**14**	184	44	55	45	14	56	82	63				
**15**	163	84	118	74								
**16**	366	55	62	55								
**17**	85	93	95	92								
**18**	87	46	53	51								
**19**	153	57	50	65								
**20**	480	75	94	83								
**21**	118	99	114	87								
**22**	121	102	116	106								
**23**	126	40	56	48								
**24**	39	74	83	71								
**25**	144	90	89	86								

Abbreviations: age and follow-up in months; TIQ, total Intelligence Quotient; VIQ, verbal Intelligence Quotient; PIQ, performance Intelligence Quotient
