# Supplementary material for: Overall cognitive profiles in patients with GLUT1 Deficiency Syndrome
Source: Brain Behav. 2019 Feb 4;9(3):e01224. doi: 10.1002/brb3.1224 (PMC6422708; doi:10.1002/brb3.1224)
Supplement: Supplementary file 2 [file BRB3-9-e01224-s002.docx]

Supplementary Table B. T0 IQ subtests expressed in standard scores in GLUT1DS population

| ID  patient | TIQ | VIQ | PIQ | Picture  Completion | Information | Coding-digit  symbol | Similarities | Picture  Arrangement | Aritmethic | Block  design | Vocabulary | Visual  puzzle | Comprehension |
| --- | --- | --- | --- | --- | --- | --- | --- | --- | --- | --- | --- | --- | --- |
| 1 | 99 | 104 | 98 | **9** | **10** | **7** | **10** | **10** | **10** | **10** | **11** | **7** | **12** |
| 3 | 43 | 54 | 45 | **1** | **3** | **1** | **5** | **3** | **1** | **4** | **5** | **2** | **2** |
| 4 | 77 | 81 | 77 | **8** | **9** | **7** | **10** | **9** | **7** | **6** | **6** | **4** | **4** |
| 5 | 48 | 59 | 48 | **5** | **2** | **3** | **4** | **1** | **3** | **1** | **7** | **3** | **4** |
| 6 | 66 | 63 | 76 | **7** | **7** | **9** | **7** | **8** | **2** | **2** | **3** | **7** | **4** |
| 7 | 59 | 76 | 71 | **6** | **7** | **4** | **6** | **7** | **7** | **6** | **8** | **7** | **4** |
| 8 | 45 | 45 | 45 | **2** | **1** | **2** | **2** | **2** | **2** | **1** | **2** | **1** | **1** |
| 9 | 79 | 94 | 89 | **7** | **9** | **9** | **7** | **9** | **10** | **9** | **10** | **8** | **10** |
| 10 | 50 | 65 | 45 | **5** | **4** | **2** | **3** | **1** | **2** | **1** | **1** | **1** | **3** |
| 11 | 51 | 59 | 54 | **7** | **5** | **2** | **4** | **3** | **6** | **4** | **5** | **3** | **3** |
| 12 | 63 | 75 | 59 | **5** | **3** | **5** | **8** | **5** | **9** | **1** | **4** | **5** | **8** |
| 13 | 52 | 56 | 58 | **4** | **2** | **4** | **6** | **2** | **2** | **1** | **6** | **1** | **1** |
| 14 | 44 | 55 | 45 | **2** | **2** | **3** | **4** | **2** | **1** | **1** | **5** | **3** | **5** |
| 15 | 84 | 118 | 74 | **6** | **13** | **2** | **13** | **8** | **11** | **7** | **14** | **8** | **12** |
| 16 | 55 | 62 | 55 | **8** | **3** | **3** | **4** | **3** | **6** | **3** | **4** | **3** | **5** |
| 17 | 93 | 95 | 92 | **8** | **9** | **7** | **11** | **10** | **8** | **10** | **9** | **11** | **9** |
| 18 | 46 | 53 | 51 | **2** | **2** | **1** | **4** | **4** | **4** | **5** | **1** | **3** | **4** |
| 19 | 57 | 50 | 65 | **4** | **2** | **4** | **3** | **6** | **4** | **5** | **2** | **6** | **2** |
| 20 | 75 | 94 | 83 | **5** | **10** | **4** | **8** | **10** | **6** | **8** | **9** | **11** | **4** |
| 21 | 99 | 114 | 87 | **10** | **12** | **5** | **13** | **11** | **11** | **3** | **10** | **12** | **14** |
| 22 | 102 | 116 | 106 | **13** | **12** | **6** | **11** | **13** | **12** | **9** | **11** | **13** | **16** |
| 23 | 40 | 56 | 48 | **2** | **5** | **4** | **2** | **3** | **5** | **3** | **2** | **1** | **4** |
| 24 | 74 | 83 | 71 | **5** | **9** | **6** | **5** | **6** | **6** | **7** | **10** | **6** | **8** |
| 25 | 90 | 89 | 86 | **7** | **8** | **8** | **9** | **8** | **9** | **10** | **7** | **6** | **9** |
